# Sensitive and quantitative determination of short-chain fatty acids in human serum using liquid chromatography mass spectrometry

**DOI:** 10.1007/s00216-021-03589-w

**Published:** 2021-08-11

**Authors:** Armaghan Shafaei, Veronica Vamathevan, Jessica Pandohee, Nathan G. Lawler, David Broadhurst, Mary C. Boyce

**Affiliations:** 1 Centre for Integrative Metabolomics and Computational Biology, School of Science, Edith Cowan University, Joondalup, WA 6027 Australia; 2 Centre for Crop and Disease Management, School of Molecular and Life Sciences, Curtin University, Bentley, WA 6102 Australia; 3 Australian National Phenome Centre, Computational and Systems Medicine, Health Futures Institute, Murdoch University, Perth, WA 6150 Australia

**Keywords:** SCFA, High-resolution mass spectrometry, LC-MS/MS, Serum

## Abstract

Short-chain fatty acids (SCFAs) are increasingly being monitored to elucidate the link between gut health and disease. These metabolites are routinely measured in faeces, but their determination in serum is more challenging due to their low concentrations. A method for the determination of eight SCFAs in serum is described here. High-resolution mass spectrometry and gas chromatography were used to identify the presence of isomeric interferences, which were then overcome through a combination of chromatographic separation and judicious choice of MS fragment ion. The SCFAs were derivatised to form 3-nitrophenylhydrazones before being separated on a reversed-phase column and then detected using liquid chromatography tandem mass spectrometry (LC-QQQ-MS). The LODs and LOQs of SCFAs using this method were in the range 1 to 7 ng mL^−1^ and 3 to 19 ng mL^−1^, respectively. The recovery of the SCFAs in serum ranged from 94 to 114% over the three concentration ranges tested.

**Supplementary Information:**

The online version contains supplementary material available at 10.1007/s00216-021-03589-w.

## Introduction

Short-chain fatty acids (SCFAs) are the end products of fermentation of dietary fibre by anaerobic intestinal microbiota. As humans lack the enzymes to degrade the bulk of dietary fibres, nondigestible carbohydrates pass the upper gastrointestinal tract unaffected and are fermented in the caecum and the large intestine by the anaerobic caecal and colonic microbiota. Fermentation results in multiple groups of metabolites including the SCFAs [1]. SCFAs are saturated aliphatic organic acids that consist of one to six carbons of which acetic acid (C2), propionic acid (C3), and butyric acid (C4) are the most abundant (95%).

The SCFAs produced in the caecum/large intestine are absorbed in the colon and either utilised in colonocytes or transported via the portal vein to reach the blood circulation and other organs. Transported SCFAs act as substrates or signal molecules that provide a link between the diet, the microbiota, and the host, and mediate health benefits locally in the gut and at the systemic level [2]. The amount and type of fibre consumed have dramatic effects on the composition of the intestinal microbiota and consequently on the type and amount of SCFAs produced.

In humans, the effect of dietary fibre intake has been studied by measuring the SCFA concentrations in faeces and calculating the total rate of SCFA excretion. Higher concentrations of SCFAs in faeces have been positively associated with a higher dietary intake of fibre [3]. However, faecal SCFA concentrations do not reflect the concentration and production rate of SCFAs in the intestine or the level of absorption of SCFAs by the host. The distinct relationship between the level of faecal SCFAs, the composition, and the ratio of the intestinal microbiota and health is unclear [4]. To gain more understanding of the link between nutritional disorders, gut microbiota, and gut microbiota–derived metabolites, it is important to accurately measure both circulating/blood and faecal SCFAs.

SCFA concentrations in the colon and faeces are relatively easy to quantify as intestinal concentrations are in the millimolar range. However, SCFA concentrations in plasma and serum are considerably lower and range between 50 and 100 μmol L^−1^ for acetate and 0.5–10 μmol L^−1^ for propionate and butyrate [5]. Thus, the measurement of SCFAs in serum at these concentrations is more complicated and prone to interferences.

Traditionally, gas chromatography (GC) is the technique of choice for the analysis of SCFAs [5–8]. However, recently, several LC-MS methods have been reported for the analysis of SCFAs [5, 9–13]. The SCFAs are typically derivatised as the low molecular weight of the native SCFAs makes them susceptible to interference when using MS detection, and also results in low recovery due to their volatility. Derivatisation has the added advantage of reducing the polarity of the SCFAs and making them more amenable to retention on a reversed-phase column.

Han et al. (2015) reported a novel derivatisation reagent, 3-nitrophenylhydrazine (3-NPH), for the sensitive detection of SCFAs by LC-MS. The method was fully validated, and the authors reported good sensitivity and importantly excellent chemical stability of the derivatives [9]. As greater detection sensitivity is required for serum and plasma samples compared to faecal samples, Zeng and Cao (2018) investigated several derivatisation reagents to determine which one provided maximum analyte sensitivity and optimal separation of the SCFAs and ketone bodies in mouse serum from interfering structural isomers [10]. While they recommended the use of o-benzylhydroxylamine (O-BHA) as a derivatising reagent for the simultaneous determination of SCFAs and ketone bodies, they did report superior sensitivity for the SCFAs when derivatised with 3-NPH compared to O-BHA. Song et al. (2019) also used LC-MS to monitor the concentrations of SCFAs in mouse plasma but used 4-acetamido-7-mercapto-2,1,3-benzoxadiazole as the derivatising reagent. While the derivatisation method had the advantage of being completed at room temperature, the excessive chromatographic run time of 65 min for baseline resolution of the isomeric structures was a serious limitation [13]. Therefore, in this work, we chose to use 3-NPH to convert SCFAs to their 3-nitrophenylhydrazones under optimised conditions for their accurate quantification in human serum using LC-MS/MS. Additionally, we report the use of GC and LC both coupled to a high-resolution mass spectrometry as orthogonal methods that use fundamentally different principles (detection or separation) to inform the development of an LC-MS/MS method.

## Materials and methods

### Chemicals and reagents

LC-MS-grade water and acetonitrile (ACN) were purchased from Thermo Fisher Scientific (Sydney, Australia). Butyric acid-D_7_ was purchased from Cambridge Isotope Laboratories (Cambridge, MA, USA). Acetic acid, propionic acid, isobutyric acid, butyric acid, isovaleric acid, valeric acid, 4-methyl valeric acid, hexanoic acid, ^13^C_2_-acetic acid, 2-ethylbutyric acid, methyl tert-butyl ether (MTBE), 3-nitrophenylhydrazine hydrochloride (3-NPH.HCl), 1-ethyl-3-(3-dimethylaminopropyl) carbodiimide hydrochloride (EDC.HCl), and pyridine were all purchased from Sigma-Aldrich (Sydney, Australia).

### Preparation standards

Stock solutions of acetic acid, propionic acid, isobutyric acid, butyric acid, isovaleric acid, valeric acid, 4-methylvaleric acid, and hexanoic acid were prepared in 20% methanol/water at concentrations of 10.5, 7.425, 1.455, 1.455, 9.6, 2.775, 2.769, and 2.79 mg mL^−1^, respectively. Mixed calibration standard solutions (eight) in the range 0.015–1 μg mL^−1^ for valeric acid, 4-methylvaleric acid, and hexanoic acid; 0.0375–2.5 μg mL^−1^ for propionic acid, isobutyric acid, and butyric acid; 0.15–10 μg mL^−1^ for isovaleric acid; and 0.375–25 μg mL^−1^ for acetic acid were prepared in water. Three QC standards at 0.015, 0.1, and 1 μg mL^−1^ for valeric acid, 4-methylvaleric acid, and hexanoic acid; 0.0375, 0.25, and 2.5 μg mL^−1^ for propionic acid, isobutyric acid, and butyric acid; 0.15, 1, and 10 μg mL^−1^ for isovaleric acid; and 0.375, 2.5, and 25 μg mL^−1^ for acetic acid were also prepared in water.

The internal standards were prepared in water at a concentration of 1 mg mL^−1^. Aliquots (2 mL) were stored at − 80 °C. An internal standard solution containing the labelled internal standards (^13^C_2_-acetic acid (35 μg mL^−1^), D_7_-butyric acid (3.84 μg mL^−1^), and 2-ethyl butyric acid (1 μg mL^−1^)) were prepared in LC-MS-grade acetonitrile.

### Serum samples

Briefly, venous blood samples were obtained from healthy volunteers from the antecubital vein and collected in SST vacutainer tubes (Becton Dickinson, NJ, USA). Serum samples were allowed to clot for at least 30 min at room temperature, before subsequently being centrifuged at 1300g for 15 min. The supernatant was then immediately aliquoted and stored at − 80 °C.

### Sample preparation for LC-MS analysis

Serum was thawed and 200 μL was transferred to a 15-mL polypropylene centrifuge tube. The ACN solution containing internal standards (600 μL) was added to the thawed serum samples, and the samples were allowed to sit on ice for 20 min. The ACN layer was separated by centrifuging the samples at 4 °C (300g × 2 min) and transferred to a separate 15-mL centrifuge tube, and 200 μL water was added to the solution. The SCFAs were derivatised to alkyl hydrazones by adding 600 μL of 200 mM 3-nitrophenylhydrazine hydrochloride (3-NPH-HCL) solution in 50% acetonitrile/water and 600 μL of 120 mM 1-ethyl-3-(3-dimethylaminopropyl) carbodiimide hydrochloride (EDC.HCl) containing 6% pyridine solution in 50% acetonitrile/water. The samples were heated at 40 °C on a Reacti-block (Hangzhou Ruicheng, China) for 30 min and then placed immediately on ice. The SCFAs were then extracted with two sequential aliquots of MTBE (5.0 mL and 4.0 mL). On each addition of MTBE, the solution was gently mixed and then centrifuged at 4 °C at (300g × 2 min). The combined MTBE extracts were dried under nitrogen at room temperature, reconstituted with 200 μL of 10% acetonitrile/water, and filtered through 3-kDa cut-off filters.

Calibration standards, QC standards, and extraction blanks (water replaced the serum) were prepared using the same procedure as for the experimental samples.

### Sample preparation for GC-HRAM-MS analysis

Serum samples were thawed and 200 μL of each sample was transferred to separate 15-mL polypropylene tubes and stored on ice. Ultra-high-purity water (200 μL) was used for a blank sample. Internal standards were added to the samples: 57 μL of ^13^C_2_-acetic acid solution (35 μg mL^−1^); 83 μL butyric acid-D_7_ (3.84 μg/mL); and 63 μL 2-ethylbutyric acid (0.8 μg mL^−1^) and mixed well. The samples were then de-proteinated by adding 50 μL of a 10% (w/w) sulfosalicylic acid solution prepared in water. The samples were then capped and left on ice for 20 min to cool and minimise possible losses of the released volatile SCFAs.

The SCFAs were extracted from the de-proteinated serum samples with 3.0 mL cold MTBE (refrigerated at 4 °C). The samples were vortexed and then centrifuged (300g × 2 min) to separate the layers. The MTBE layer was transferred to a clean tube containing 50 μL of cold 0.2 M aqueous sodium hydroxide solution to back extract the SCFAs into the aqueous layer. After vortexing and centrifuging, the MTBE layer was discarded. Cold phosphoric acid solution (30 μL, 1.0 M) was then added to the samples and vortexed to mix. The samples were transferred to cold 2 mL GC vials fitted with 200-μL tapered glass inserts. The samples were diluted 1:5 or 1:8 with cold high-purity water prior to analysis by GC–high-resolution accurate-mass (HRAM)–MS (GC-HRAM-MS).

### LC-HRAM-MS analysis

A Thermo Scientific Ultimate 3000 Liquid Chromatography coupled to a Thermo Scientific Q Exactive Focus Orbitrap Mass Spectrometer was used to quantify the SCFAs in serum. The pooled serum samples were separated on a Thermo Hypersil aQ column and an ACE C18-PFP column, both having dimensions of 100 mm × 2.1 mm ID and packed with 1.7 μm particles. Water (A) and ACN (B) were used for analyte separation. The initial mobile phase conditions were 99% A and 1% B, held for 2 min. Solvent A was reduced to 90% over 2 min, and then further reduced to 10% over 1 min and held for 5 min before returning to initial conditions. The flow rate was 0.2 mL min^−1^ and column temperature was maintained at 30 °C.

An ACE C18-AR column (100 mm × 2.1 mm ID; 1.7 μm particle diameter) was also used to separate the SCFAs using water (A) and acetonitrile (B)–based mobile phase. Initial conditions were 80% A, held for 0.5 min. B was increased to 30% over 6 min and then to 40% over 4.5 min. The mobile phase was then increased to 95% B over 1.5 min and held for 2 min. The mobile phase was then returned to starting conditions. The column was maintained at 45 °C and the flow rate 0.3 mL min^−1^. The sample injection volume was 2 μL.

Full-scan, single ion monitoring (SIM) and parallel reaction monitoring (PRM) analyses were performed using a Q Exactive Focus mass spectrometer. Electrospray ionisation in the negative ion mode was used with a spray voltage of 2500 V, an auxiliary gas temperature of 350 °C, and a capillary temperature of 350 °C. Full details of the MS settings for all three modes are provided in the Supplementary information.

### GC-HRAM-MS analysis

A Thermo Fisher Scientific Q Exactive GC-MS instrument was used to quantify SCFAs in serum extracts. A robotic arm (Thermo Scientific ™ TriPlus™ RSH autosampler) was used to inject 1 μL of sample into a split/splitless (SSL) injector at 230 °C using a 1:5 split flow on a Thermo Fisher Scientific™ TRACE™ 1310 GC. Helium was used as the carrier gas at a flow rate of 1.0 mL min^−1^ with chromatographic separation on a Thermo Fisher Scientific TG-WAXMS A column (30 m length × 0.25 mm inner diameter × 0.25 μm film thickness column). The initial oven temperature was held at 50 °C for 30 s, followed by a 30 °C min^−1^ temperature ramp to 110 °C. The oven temperature was then ramped at 5 °C min^−1^ to 135 °C and then at 20 °C min^−1^ to a final temperature of 230 °C and held for 2 min. The transfer line temperature was set to 230 °C. Electron ionisation (EI) at 65 eV (positive polarity) and an emission current of 50 μA with an ion source temperature of 250 °C were used in all experiments. A filament delay of 5.0 min was used, and the mass spectrometer was operated at a resolution of 120,000 (FWHM at *m*/*z* 200). Time-scheduled data acquisition for the SCFAs was performed by 2 experiments: SIM for acetic acid (5.5 to 7 min) and PRM (7 to 11 min) for the remaining SCFAs. The ions monitored for SIM analysis and the precursor-product transitions monitored in PRM in the inclusion list are summarised in Table S1. A collision energy of 10 eV was used for all PRM transitions, and nitrogen was used as the collision gas. A mass isolation window of 1.0 *m*/*z* was used, and the maximum injection time was set to “auto” in both SIM and PRM experiments.

### LC-QQQ-MS analysis

A Thermo Scientific Ultimate 3000 Liquid Chromatography coupled to a Thermo Scientific TSQ Quantiva Triple Quadrupole Mass Spectrometer was used for the validation study. Detection was performed in negative mode (2500 V), and the analytes were ionised by electrospray and monitored in multiple reaction monitoring (MRM) mode. The LC conditions were as described above for the ACE C18-AR column. The MS conditions were gases (arbitrary units) sheath 50, auxiliary 10, sweep 1, ion transfer tube temperature 325 °C, and vaporiser temperature 350 °C. The ion transitions and collision energies are presented in Table S2.

### Method validation

Method validation was performed on LC-QQQ and in accordance with ICH and IUPAC guidelines [14, 15].

### Linearity, limit of detection, and limit of quantification

Linearity was determined by constructing calibration curves over the concentration range (0.015–1 μg mL^−1^ for valeric acid, 4-methylvaleric acid, and hexanoic acid; 0.0375–2.5 μg mL^−1^ for propionic acid, isobutyric acid, and butyric acid; 0.15–10 μg mL^−1^ for isovaleric acid; and 0.375–25 μg mL^−1^ for acetic acid) using each of the eight prepared calibration standards (see “Preparation standards”). The limit of detection (LOD) and limit of quantification (LOQ) of the method were determined as a signal to noise ratio (*S*/*N*) of 3 and 10 of diluted standard solution, respectively.

### Precision, trueness, and accuracy

Precision and trueness of the method were determined by an intra-day and inter-day analysis of the calibration standards. The calibration standards were injected 6 times per day (intra-day) and 1 time per day for 6 consecutive days (inter-day). The resulting peak area was used to calculate the coefficient of variation (% CV) and thus precision. The calculated mean concentration relative to the nominal concentration was used to reveal trueness (% bias). Moreover, the relative standard deviation (% RSD) of the obtained retention times for each analyte for 12 injections was calculated to predict the retention time repeatability of the method.

The accuracy of the method was evaluated by a recovery test. In brief, three pooled serum samples (200 μL) were spiked with 20 μL of mixed standard solutions at low (LQC), medium (MQC), and high (HQC) levels of 0.058, 0.232, and 0.465 μg mL^−1^ for hexanoic acid; 0.058, 0.231, and 0.462 μg/mL for 4-methyl valeric acid; 0.124, 0.495, and 1.238 μg mL^−1^ for propionic acid; 0.462, 2.308, and 4.615 μg mL^−1^ for isovaleric acid; 0.058, 0.233, and 0.465 μg/mL for valeric acid; 0.121, 0.485, and 1.213 μg/mL for isobutyric acid; 0.120, 0.480, and 1.2 μg mL^−1^ for butyric acid; and 0.525, 5.250, and 13.125 μg mL^−1^ for acetic acid, respectively. The baseline (unspiked) and spiked serum samples at each concentration were analysed, and the percentage of recovery was calculated according to Eq. (1), where *C*_baseline_ is the calculated unspiked analyte concentration, *C*_recovered_ is the calculated spiked analyte concentration, and *C*_spiked_ is the absolute concentration of spiked standard added to the sample.

1 $$ \mathrm{Relative}\ \mathrm{recovery}=\frac{C_{\mathrm{recovered}}-{C}_{\mathrm{baseline}}}{C_{spiked}}\times 100\% $$

## Results and discussion

### Chromatographic separation of SCFA

Pooled serum samples were derivatised to form 3-nitrophenylhydrazones, and first separated on a Thermo Hypersil aQ column, and analysed using high-resolution Orbitrap mass spectrometry in three modes: full scan, SIM, and PRM. For FS and SIM analysis, the precursor ion for each analyte was used for quantification (Table S1). For PRM analysis, at least two product ions were monitored and the collision energy which gave the optimal signal for these product ions was experimentally determined and adopted (Table S1).

The concentration of the SCFA in the pooled samples, separated on the Hypersil aQ column and analysed by HRAM-MS, was determined using a one-point calibration using a standard solution with SCFA concentrations that closely matched the expected concentration in serum and using labelled and non-labelled internal standards (^13^C_2_-acetic acid, butyric acid-D_7_, and 2-ethylbutyric acid). There was good agreement between the three modes for all analytes, with the exception of propionic acid (Table 1). The agreement between FS and SIM for propionic acid is expected as both modes monitor the precursor ion. The lower concentration reported using the PRM mode, when compared to SIM and FS, suggests there is an isobaric interference with the precursor ion resulting in elevated results for SIM and FS. In PRM mode, quantification is determined using a fragment ion, which is monitored at high resolution providing extra selectivity and hence resolution from coeluting background ions.

**Table 1:** Comparison of different methods (full scan, PRM, and SIM on LC-HRAM-MS and PRM on GC-HRAM-MS) for analysis of SCFAs in different pooled serum samples (*n* = 2). SCFAs were separated on a Thermo Hypersil aQ column (LC-HRAM-MS) and on a Thermo TG-WAX column (GC-HRAM-MS)

Analyte	ISTD	Sample	LC-HRAM-MS						GC-HRAM-MS	
			PRM		FS		SIM		PRM	
			Concentration (ng mL^−1^)	SD	Concentration (ng mL^−1^)	SD	Concentration (ng mL^−1^)	SD	Concentration (ng mL^−1^)	SD
Acetic acid	^13^C_2_-Acetic acid	Pool 1	14,627	558	14,782	31.2	15,110	111	10,064	259
		Pool 2	14,933	273	14,872	35.3	15,303	530	12,344	1532
Propionic acid	Butyric acid-D_7_	Pool 1	1271	6.9	2060	90.8	2218	82.6	1188	58.1
		Pool 2	1200	13.9	1861	22.3	2085	52.1	1115	94.3
Isobutyric acid	Butyric acid-D_7_	Pool 1	1761	9.4	1749	49.0	1736	18.1	2161	125
		Pool 2	1717	12.8	1695	9.3	1716	50.3	2194	173
Butyric acid	Butyric acid-D_7_	Pool 1	1387	10.6	1420	11.4	1410	31	1787	137
		Pool 2	1479	25.9	1331	16.4	1354	33.7	1654	141
Isovaleric acid	Butyric acid-D_7_	Pool 1	2766	51.4	2874	37.2	2656	23.7	4387	330
		Pool 2	3274	23.1	3334	54.0	3171	78.5	5144	952
Valeric acid	2-Ethylbutyric acid	Pool 1	107	1.1	78.2	1.6	78.7	4.0	124	11.3
		Pool 2	108	1.6	91.2	1.9	89.6	0.9	115	17.1
4-Methylvaleric acid	2-Ethylbutyric acid	Pool 1	15.7	1.3	23.9	1.8	13.9	2.4	23.1	1.9
		Pool 2	15.7	1.0	21.6	0.8	13.2	0.0	22.4	1.3
Hexanoic acid	2-Ethylbutyric acid	Pool 1	826	22.3	826	8.0	800	131	912	32.9
		Pool 2	761	9.3	777	21.8	790	8.6	916	73

**Fig. 1:**
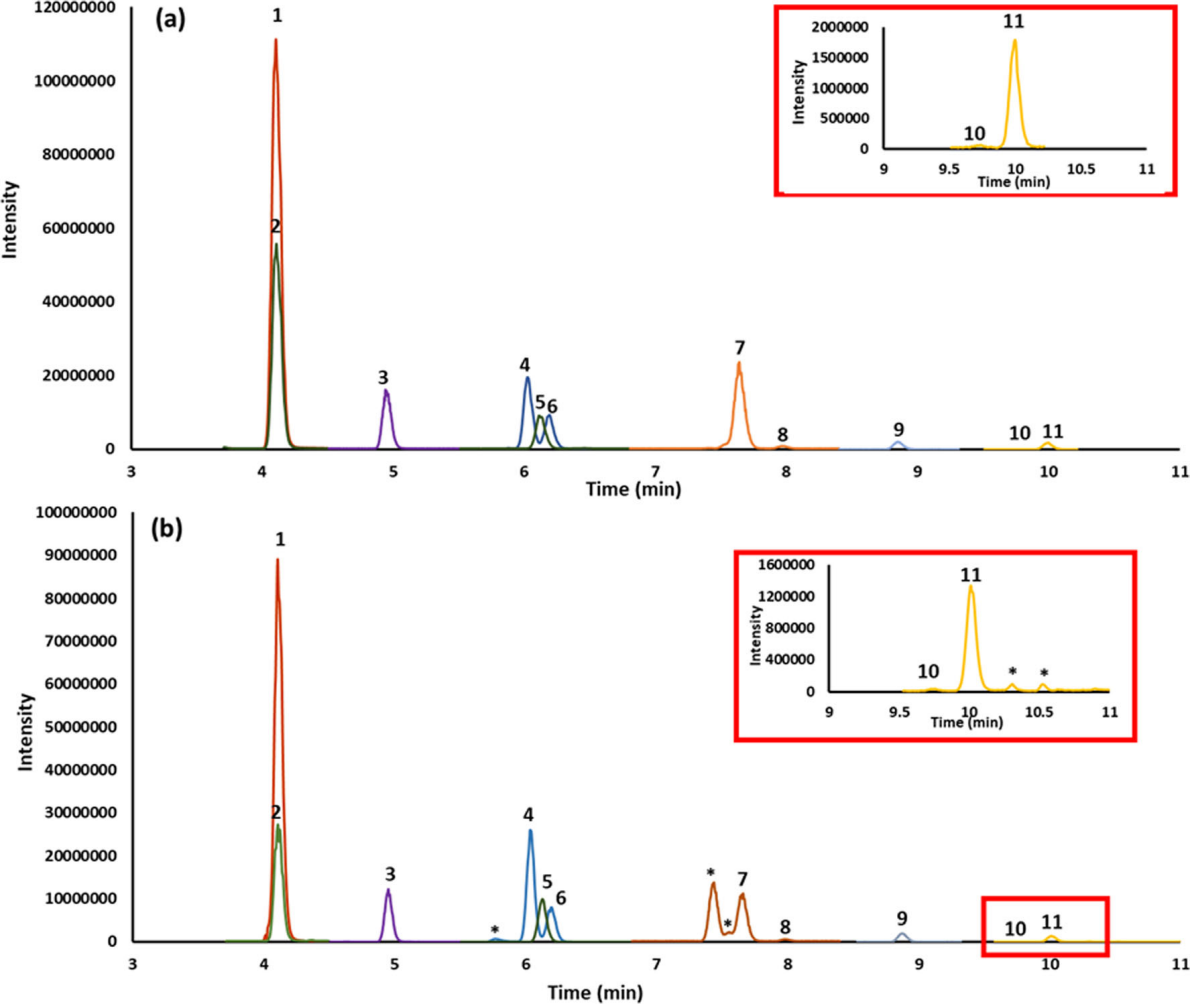
Overlay of extracted ion chromatograms of (1) acetic acid, (2) ^13^C_2_-acetic acid, (3) propionic acid, (4) isobutyric acid, (5) butyric acid-D_7_, (6) butyric acid, (7) isovaleric acid, (8) valeric acid, (9) 2-ethyl butyric acid, (10) 4-methyl valeric acid, and (11) hexanoic acid of **a** standard mix and **b** pooled serum sample both acquired in PRM mode on a Thermo Hypersil aQ column (* interference)

**Fig. 2:**
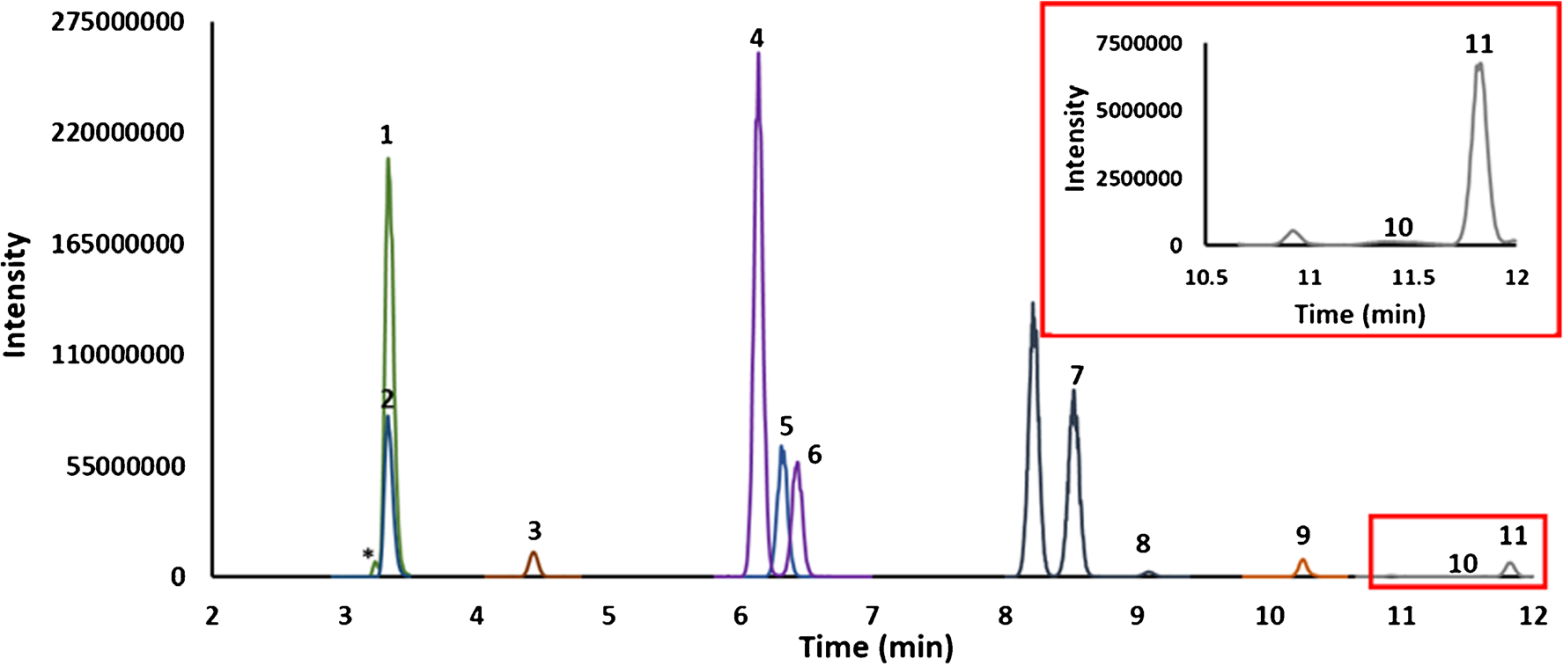
Overlay of extracted ion chromatograms of (1) acetic acid, (2) ^13^C_2_-acetic acid, (3) propionic acid, (4) isobutyric acid, (5) butyric acid-D^7^, (6) butyric acid, (7) isovaleric acid, (8) valeric acid, (9) 2-ethyl butyric acid, (10) 4-methyl valeric acid, and (11) hexanoic acid of pooled serum sample separated on an ACE C18-AR column in PRM (* acetic acid interference)

The chromatographic method using the Hypersil aQ column resolved the SCFAs (Fig. 1). Several isobaric or isomeric compounds, and specifically those relating to the butyric and valeric acids, present in the serum samples were also resolved (Fig. 1). The fragmentation pattern and predicted elemental composition for these interferences indicated they were likely isomeric (see Supplementary information for MS/MS fragmentation pattern and elemental composition). A slight shoulder on the acetic acid peak for some of the pooled serum samples indicated the presence of an unresolved interference. In PRM mode, different fragment ions were checked to determine if selecting and monitoring an alternative fragment ion would remove this interference. This approach was not successful, so chromatographic resolution of the interference was necessary. Several columns were trialled to resolve acetic acid from the interfering peak. An ACE C18-PFP column with the same acetonitrile/water gradient program resolved the interference for acetic acid; however, there was loss of resolution for isovaleric and its interferences. An ACE C18-AR column with a modified gradient consisting of water and acetonitrile as mobile phase successfully resolved acetic acid from its interference, and at the same time maintained resolution of the interferences for butyric acid and isovaleric acid (Fig. 2). Having resolved the acetic acid interference, it was then possible to determine the potential contribution of the interference to the acetic acid peak; it varied from 0 to 12% depending on the sample.

**Fig. 3:**
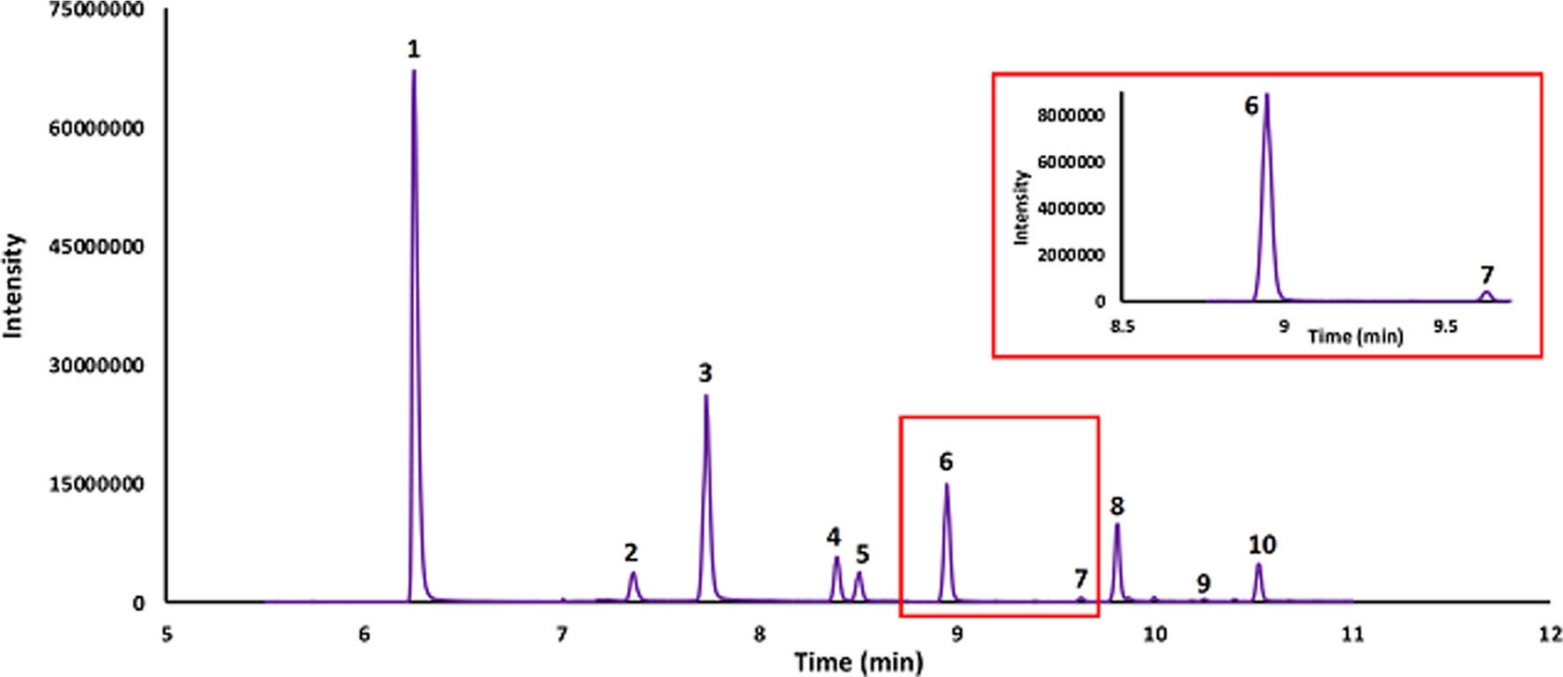
Total ion chromatograms of (1) acetic acid, (2) propionic acid, (3) isobutyric acid, (4) butyric acid-D_7_, (5) butyric acid, (6) isovaleric acid, (7) valeric acid, (8) 2-ethyl butyric acid, (9) 4-methyl valeric acid, and (10) hexanoic acid in pooled serum sample separated on a Thermo TG-WAX column in GC-HRAM-MS

As gas chromatography has traditionally been used to determine SCFAs, particularly for faecal material, the pooled serum samples were also separated using GC coupled to a HRAM-MS which was operated in SIM (acetic acid) or PRM mode (all other analytes) (Fig. 3). The analytes were quantified as for LC-HRAM-MS and the data are presented in Table 1. As an orthogonal method, this GC experiment was completed to determine if there were significant unresolved interferences in the LC method. Elevated concentrations of SCFA in the LC data when compared to the GC data would suggest unresolved interferences. There was generally good agreement between the LC-HRAM-MS (PRM mode) and the GC-HRAM-MS (PRM mode) methods (± 20%), particularly with respect to the relative concentrations of the SCFAs in a given sample. However, there was a large discrepancy between the two techniques for isovaleric acid and to a lesser extent for isobutyric acid, where the GC method reported elevated concentrations when compared to the LC method. This suggested that the interferences visible and resolved in LC-MS (Fig. 1) are unresolved in GC and contribute to the analyte signal. No attempt was made to resolve the interferences in the GC method as the aim of this work was to develop an LC-MS method.

**Fig. 4:**
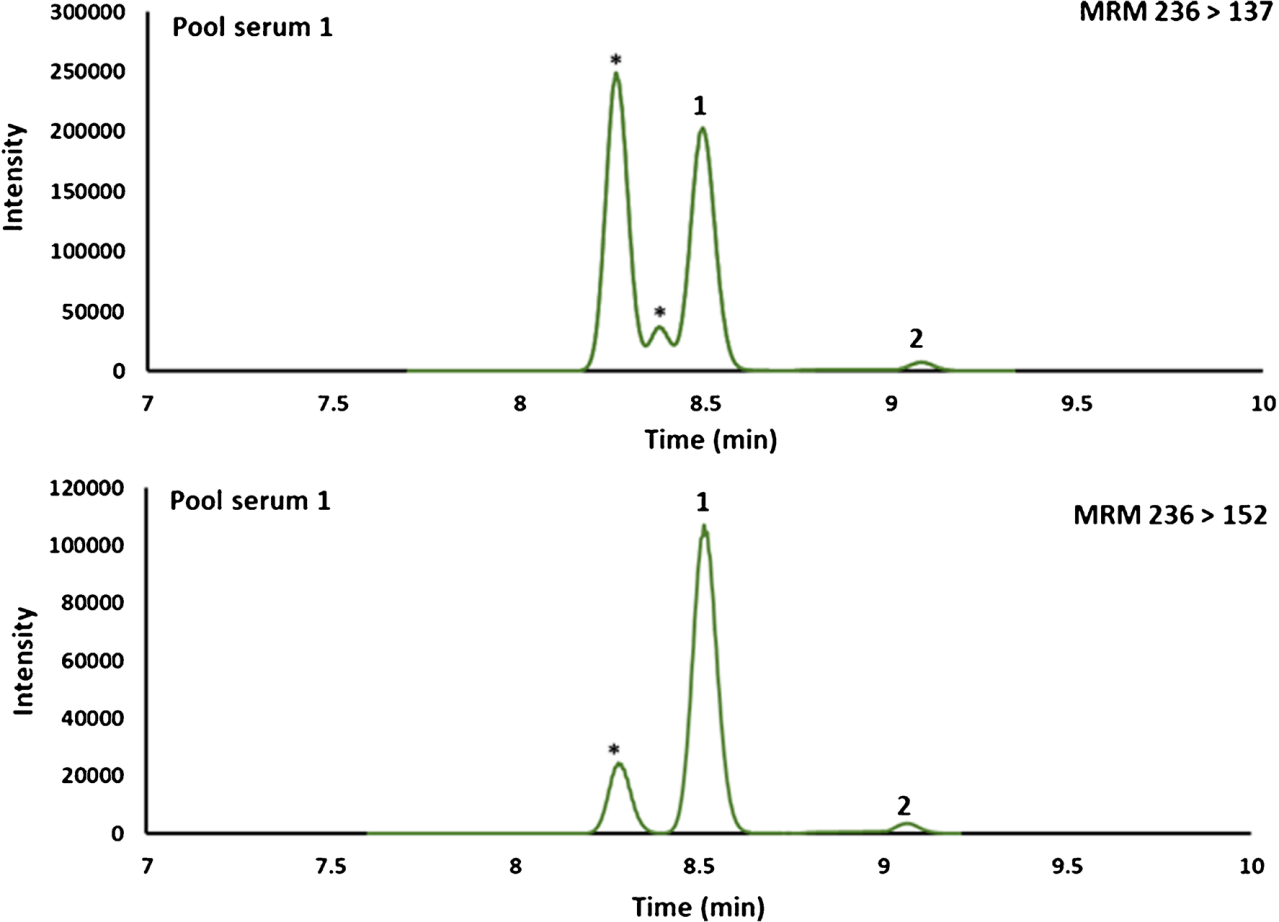
Extracted ion chromatograms of MRM transitions (236 > 137 and 236 > 152) monitored for (1) isovaleric acid and (2) valeric acid on an ACE C18-AR column in MRM (* interference)

The use of high-resolution mass spectrometry and orthogonal methods when developing methods for analytes in complex matrices such as serum is informative. However, for routine analysis, tandem mass spectrometry is preferred and more widely available. The application of this method to a low-resolution MS instrument, a triple quadrupole, was explored. The SCFA in five pooled samples were quantitatively determined using an eight-point calibration curve by both HR-LC-MS in PRM mode and LC-HRAM-MS in MRM mode after separation on the ACE C18-AR column. A similar quantitative ion was selected for most SCFAs in PRM and MRM with the exception of isovaleric acid and valeric acid. The transition 236 ➔ 137 for isovaleric acid showed contribution from an unresolved interference for some samples (Fig. 4). Therefore, transition 236 ➔ 152 where the interference was not detected was used as the quantitative ion for the tandem mass spectrometry method (Fig. 4).

There was excellent agreement (95–106%) between the data obtained from PRM (HRAM instrument) and MRM (tandem MS instrument) modes for acetic, propionic, and isobutyric acids (Table S5). For butyric acid and isovaleric acid, there was good agreement (94–117%). However, for the minor SCFAs, valeric acid and 4-methylvaleric acid were present at lower concentrations, and typically between 10 and 100 ng mL^−1^, the agreement was less with the percentage agreement between 75 and 131% (Table S5). This comparison of HRAM vs tandem MS after separation on the ACE C18-AR column was repeated but with 11 individual serum samples, and again, excellent agreement was obtained for the SCFAs with the exception of 4-methylvaleric acid, where the agreement was lower at 71–108% due to its presence in serum at low concentrations (Table 2). The advantage of using HRAM-MS to inform the development of a robust method on a more accessible instrument has been demonstrated.

**Table 2:** Comparison of MRM mode in LC-QQQ and PRM mode in LC-HRAM-MS for quantitative analysis of SCFAs in 11 serum samples. SCFAs were separated on an ACE C18-AR column

Target compound	Concentration range LC-QQQ-MS		Concentration range LC-HRAM-MS		Agreement (%)
	ng mL^−1^	nM	ng mL^−1^	nM	
Acetic acid	3170–12,782	52,788–212,849	3100–13,015	51,622–216,729	86.4–109
Propionic acid	360–1136	4860–15,335	350–1156	4725–15,605	93.7–103
Isobutyric acid	90–1241	1021–14,085	100–1211	1135–13,745	90–105
Butyric acid	122–1247	1385–14,153	124–1247	1407–14,153	93.9–109
Isovaleric acid	23.5–3992	230–39,086	23.5–3932	230–38,499	92.7–110
Valeric acid	20–100	196–979	21–106	206–1038	91.5–110
4-Methylvaleric acid	3.8–12	33–103	3.7–14	31.8–120.5	71.4–108
Hexanoic acid	30–391	258–3366	27–385	232–3314	95.7–111

The LC-MS method was just 15 min long comparing favourably with other published methods: Song et al. (2019) separated the SCFAs in 35 min [13], while Wei et al. (2020) reported a run time of 14 min [11] and Zeng et al. (2018) reported a run time of just 6 min [10]. The stability of the SCFA-3-NPH derivatives was previously established by Han et al. (2015) and was therefore not investigated here [9].

### Tandem MS method validation

#### Linearity, LOD, and LOQ

An eight-point calibration curve was plotted for each analyte using the ratio of the peak area of the analyte/peak area of IS versus the ratio of the concentration of the analyte/concentration of IS. Good linearity was obtained for all analytes over the evaluated concentration range (0.015–1 μg mL^−1^ for valeric acid, 4-methylvaleric acid, and hexanoic acid; 0.0375–2.5 μg mL^−1^ for propionic acid, isobutyric acid, and butyric acid; 0.15–10 μg/mL for isovaleric acid; and 0.375–25 μg mL^−1^ for acetic acid) with correlation coefficients greater than *r*^2^ = 0.999 for all analytes. The limits of detection (LODs) and limits of quantitation (LOQs) of the method were calculated (Table S3). The concentration range for each of the SCFA in the serum samples tested varied quite widely—often as much as 100-fold. For example, the ranges reported in the 11 serum samples tested for isobutyric acid and isovaleric acid were 90–1241 ng mL^−1^ and 23–3992 ng mL^−1^ respectively. However, the LOQ of the method for these analytes (3 and 7 ng mL^−1^ for isobutyric acid and isovaleric acid respectively) was sufficient and below the lowest concentration reported. The LOQ for the method was lower than the concentration detected for all analytes and all serum samples (pooled and non-pooled) with one exception—one serum sample reported a concentration for 4-methylvaleric below its LOQ but above its LOD. Han et al. (2015) measured the 3-NPH derivatives of the SCFAs in faeces, so this work demonstrates that the derivatising reagent is also sensitive enough for serum applications, where the concentrations of the SCFAs are several orders of magnitude lower [9]. Song et al. (2019) selected 4-acetamido-7-mercapto-2,1,3-benzoxadiazole as the derivatising reagent to monitor SCFAs in mouse serum, but with LOQs typically 3–10 times higher than reported here, minor SCFAs such as valeric and isovaleric acid would not be quantifiable in human serum [13]. Wei et al. (2020) quantified the SCFAs in mouse serum after derivatisation with d_0_/d_6_-N, N-dimethyl-6,7-dihydro-5H-pyrrolo [3,4-d] pyrimidine-2-amine using HRAM-MS operating in PRM mode and reported LOQs in the range 0.5–4 fg on column (for a 10-μL sample injection) [11]. Our LOQs based on a similar injection volume compare favourable ranging from 1 to 5 fg on column. Zhang et al. (2020) recently reported a GC-MS method where the SCFAs extracted from mice serum were derivatised with BSTFA to improve sensitivity and reported LOQs in the range 100–200 ng/mL which are at least an order of magnitude higher than reported here [5].

#### Precision, trueness, and accuracy

Intra- and inter-day variations were determined by analysing eight different concentrations of calibration standards for six replicates in a single day (intra-day) and one replicate for six consecutive days (inter-day) to evaluate the precision (% CV) and trueness (% bias) of the developed analytical method. The CV ranged from 0.34 to 12.58% and the bias for both intra-day and inter-day studies was within ± 20% with the exception of propionic acid at 0.25 μg mL^−1^, where the % bias was 23.29% (Table S3).

Retention time repeatability for each analyte was determined by calculating the %RSD from the obtained retention time after 12 injections. The %RSDs were less than 0.3% and as low as 0.02%. Specifically, the %RSDs for retention time repeatability were 0.29, 0.20, 0.04, 0.06, 0.04, 0.03, 0.05, and 0.02 for acetic acid, propionic acid, isobutyric acid, butyric acid, isovaleric acid, valeric acid, 4-methyl valeric acid, and hexanoic acid, respectively.

The accuracy of the method was determined by recovery study. The recovery of the SCFAs in serum ranged from 93.97 to 113.81% in this study and over the three concentration ranges tested (Table S4) and compared very favourably with recent methods for analysis of SCFAs in serum by LC-MS. Song et al. (2019) reported recoveries ranging between 82 and 116% [13], and Wei et al. (2020) reported recoveries of between 80 and 120% [11] for the determination of SCFA in mouse serum, and using LC-MS.

## Conclusion

The focus of this work was to highlight the role of high-resolution mass spectrometry and orthogonal methods in method development particularly when the matrix is complex and the analytes are present at low concentrations. A method for the determination of SCFAs in serum is reported using a reversed-phase column for separation and MS for detection. The presence of several interferences was detected using high-resolution mass spectrometry. Chromatographic separation was necessary to resolve the interferences using a C18 column with embedded aromatic functionality (ACE C18-AR column). The HRAM-MS method was successfully transferred to a QQQ method. The latter method was validated using both pooled and individual serum samples.

## Supplementary information

ESM 1 (DOCX 165 kb)

## Data Availability

The online version contains supplementary material.
