# Supplementary material for: Sensitive and quantitative determination of short-chain fatty acids in human serum using liquid chromatography mass spectrometry
Source: Anal Bioanal Chem. 2021 Aug 11;413(25):6333–42. doi: 10.1007/s00216-021-03589-w (PMC8487878; doi:10.1007/s00216-021-03589-w)

**Supplementary Information**

Sensitive and Quantitative Determination of Short-Chain Fatty Acids in Human Serum Using Liquid Chromatography Mass Spectrometry

Armaghan Shafaei^1^, Veronica Vamathevan^1^, Jessica Pandohee^2^, Nathan G Lawler^3^, David Broadhurst^1^, and Mary C Boyce^*1^

^1^Centre for Integrative Metabolomics and Computational Biology, School of Science, Edith Cowan University, Joondalup, WA 6027, Australia.

^2^Centre for Crop and Disease Management, School of Molecular and Life Sciences, Curtin University, Bentley, WA 6102, Australia

^3^Australian National Phenome Centre, Computational and Systems Medicine, Health Futures Institute, Murdoch University, Perth, WA 6150, Australia.

^*^Corresponding author. E-mail: [m.boyce@ecu.edu.au](mailto:m.boyce@ecu.edu.au)

**HRAM-MS Conditions**

For full scan analysis, signal acquisition was performed in the mass range m/z 95-300, an orbitrap resolution of 70,000 (at m/z 200) and an automatic gain control target (AGC) of 1e^5^ and a chromatographic peak width of 6 s was specified.

SIM analysis was performed using an orbitrap resolution of 35,000 (at m/z 200), automatic gain control target (AGC) of 5e^4^ and a chromatographic peak width of 6 s was specified. The SIM method employed a multiplexing degree of 2 for acquisition of the native and the corresponding isotopically labelled internal standard with a mass isolation window of 2.0 m/z. SIM acquisition was performed using timed segments. The precursor masses acquired in the method are summarised in Table 1S

Time-scheduled PRM acquisition was performed using an orbitrap resolution of 17,500 (at m/z 200), automatic gain control target (AGC) of 5e^4^, and a chromatographic peak width of 6 s was specified. A mass isolation window of 3.0 m/z was used. The precursor masses monitored in the PRM method with the corresponding optimised collision energies used for maximum extracted ion signals are summarised in Table 1S.

**Table S1** Full scan, SIM, and PRM methods for LC-HRMS and GC-HRMS analyses of SCFAs in serum samples.

| **Analyte** | **LC-HRMS** | | | |  |  | |  | | | **GC-HRMS** | | | |
| --- | --- | --- | --- | --- | --- | --- | --- | --- | --- | --- | --- | --- | --- | --- |
|  | **FS/SIM** |  | **PRM** | |  | **SIM** |  | | **PRM** | | | |  |  |
|  | **m/z** |  | **Extracted ions (m/z)** | **Collision energy (eV)** |  | **m/z** |  | | | **Extracted ions (m/z)** | | **Collision energy (eV)** | |  |
| **Acetic acid** | 194.0560 |  | 137 | 20 |  | 60.0206 |  | | | NA | | NA | |  |
|  |  |  | 152^*^ | 12 |  |  |  | | |  |  |  |  |  |
|  |  |  | 122 | 18 |  |  |  | | |  |  |  |  |  |
| **^13^C_2_-Acetic acid** | 196.0627 |  | 152 | 12 |  | 62.0273 |  | | | NA | | NA | |  |
|  |  |  | 122 | 16 |  |  |  | | |  |  |  |  |  |
|  |  |  | 137^*^ | 17 |  |  |  | | |  |  |  |  |  |
| **Propionic acid** | 208.0717 |  | 165 | 12 |  | NA |  | | | 73.0284^*^ | | 10 | |  |
|  |  |  | 152 | 14 |  |  |  | | | 74.0362 | | 10 | |  |
|  |  |  | 137^*^ | 18 |  |  |  | | | 77.0535 | | 10 | |  |
| **Isobutyric acid** | 222.0873 |  | 137^*^ | 22 |  | NA |  | | | 73.0284^*^ | | 10 | |  |
|  |  |  | 179 | 13 |  |  |  | | | 74.0362 | | 10 | |  |
|  |  |  | 152 | 16 |  |  |  | | | 77.0535 | | 10 | |  |
| **Butyric acid** | 222.0873 |  | 137^*^ | 19 |  | NA |  | | | 73.0284^*^ | | 10 | |  |
|  |  |  | 152 | 16 |  |  |  | | | 74.0362 | | 10 | |  |
|  |  |  | 179 | 13 |  |  |  | | | 77.0535 | | 10 | |  |
| **Butyric acid-D_7_** | 229.1313 |  | 137^*^ | 23 |  | NA |  | | | 73.0284 | | 10 | |  |
|  |  |  | 186 | 13 |  |  |  | | | 74.0362 | | 10 | |  |
|  |  |  | 153 | 16 |  |  |  | | | 77.0535^*^ | | 10 | |  |
| **Isovaleric acid** | 236.1030 |  | 152^*^ | 18 |  | NA |  | | | 73.0284 | | 10 | |  |
|  |  |  | 193 | 14 |  |  |  | | | 87.0441^*^ | | 10 | |  |
| **Valeric acid** | 236.1030 |  | 193 | 14 |  | NA |  | | | 73.0284^*^ | | 10 | |  |
|  |  |  | 152^*^ | 18 |  |  |  | | | 87.0441 | | 10 | |  |
| **2-Ethylbutyric acid** | 250.1186 |  | 137^*^ | 20 |  | NA |  | | | 87.0441^*^ | | 10 | |  |
|  |  |  | 152 | 18 |  |  |  | | | 88.0519 | | 10 | |  |
| **4-Methylvaleric acid** | 250.1186 |  | 152 | 18 |  | NA |  | | | 73.0284 | | 10 | |  |
|  |  |  | 137^*^ | 23 |  |  |  | | | 74.0362^*^ | | 10 | |  |
|  |  |  | 207 | 12 |  |  |  | | | 87.0441 | | 10 | |  |
| **Hexanoic acid** | 250.1186 |  | 207 | 13 |  | NA |  | | | 73.0284 | | 10 | |  |
|  |  |  | 152 | 17 |  |  |  | | | 74.0362 | | 10 | |  |
|  |  |  | 137^*^ | 25 |  |  |  | | | 87.0441^*^ | | 10 | |  |

^*^Quan ion

**Table S2** MRM parameters used in the LC-MS/MS method for analysis of SCFAs in serum samples

| **Compound** | **Retention Time (min)** | **RT Window (min)** | **Polarity** | **Precursor (m/z)** | **Product (m/z)** | **Collision Energy (V)** | **Min Dwell Time (ms)** | **RF Lens (V)** |
| --- | --- | --- | --- | --- | --- | --- | --- | --- |
| **Acetic acid** | 3.30 | 0.6 | Negative | 194 | 137 | 18 | 123.48 | 48 |
| **Acetic acid** | 3.30 | 0.6 | Negative | 194 | 152^*^ | 10 | 123.48 | 48 |
| **^13^C_2_-Acetic acid** | 3.30 | 0.6 | Negative | 196 | 137 | 18 | 123.48 | 54 |
| **^13^C_2_-Acetic acid** | 3.30 | 0.6 | Negative | 196 | 152^*^ | 13 | 123.48 | 54 |
| **Propionic acid** | 4.40 | 0.8 | Negative | 208 | 137^*^ | 18 | 248.65 | 61 |
| **Propionic acid** | 4.40 | 0.8 | Negative | 208 | 165 | 12 | 248.65 | 61 |
| **Isobutyric acid** | 5.98 | 1.2 | Negative | 222 | 137^*^ | 20 | 81.87 | 61 |
| **Isobutyric acid** | 5.98 | 1.2 | Negative | 222 | 179 | 10 | 81.87 | 61 |
| **D_7_-Butyric acid** | 6.20 | 0.4 | Negative | 229 | 137^*^ | 21 | 81.87 | 65 |
| **D_7_-Butyric acid** | 6.20 | 0.4 | Negative | 229 | 153 | 17 | 81.87 | 65 |
| **Butyric acid** | 6.35 | 1.2 | Negative | 222 | 137^*^ | 17 | 81.87 | 51 |
| **Butyric acid** | 6.35 | 1.2 | Negative | 222 | 151 | 10 | 81.87 | 51 |
| **Isovaleric acid** | 8.48 | 1.4 | Negative | 236 | 137^*^ | 21 | 123.69 | 63 |
| **Isovaleric acid** | 8.48 | 1.4 | Negative | 236 | 152 | 16 | 123.69 | 63 |
| **Valeric acid** | 9.00 | 1.4 | Negative | 236 | 137^*^ | 21 | 123.69 | 65 |
| **Valeric acid** | 9.00 | 1.4 | Negative | 236 | 152 | 17 | 123.69 | 65 |
| **2-Ethylbutyric acid** | 10.20 | 0.8 | Negative | 250 | 107 | 30 | 82.07 | 71 |
| **2-Ethylbutyric acid** | 10.20 | 0.8 | Negative | 250 | 137^*^ | 21 | 82.07 | 71 |
| **4-Methylvaleric acid** | 11.25 | 2 | Negative | 250 | 137^*^ | 22 | 82.07 | 66 |
| **4-Methylvaleric acid** | 11.25 | 2 | Negative | 250 | 152 | 18 | 82.07 | 66 |
| **Hexanoic acid** | 11.75 | 2 | Negative | 250 | 137^*^ | 20 | 82.07 | 63 |
| **Hexanoic acid** | 11.75 | 2 | Negative | 250 | 152 | 17.3 | 82.07 | 63 |

**Table S3** LOD, LOQ, and percentage recovery of SCFAs in serum (n=3).

| **Target compound** | **LOD ng/mL** | **LOQ ng/mL** | **Concentration (µg/mL)** | | | **%Recovery** |
| --- | --- | --- | --- | --- | --- | --- |
|  |  |  | **Baseline^*^** | **Spiked** | **Measured^*^** |  |
| **Acetic acid** | 7 | 13 | 3169 ± 61.5 | 525 | 3710 ± 16.3 | 103 |
|  |  |  |  | 5250 | 8841 ± 190 | 108 |
|  |  |  |  | 13125 | 15503 ± 219 | 93.9 |
| **Propionic acid** | 6 | 19 | 359 ± 21.9 | 124 | 493 ± 19.1 | 108 |
|  |  |  |  | 495 | 863 ± 42.4 | 102 |
|  |  |  |  | 1238 | 1598 ± 127 | 100 |
| **Isobutyric acid** | 1 | 3 | 87 ± 4.2 | 121 | 225 ± 7.1 | 114 |
|  |  |  |  | 485 | 563 ± 47.4 | 98 |
|  |  |  |  | 1213 | 1398 ± 30.4 | 108 |
| **Butyric acid** | 1 | 3 | 1187 ± 18.4 | 120 | 1312 ± 12.7 | 104 |
|  |  |  |  | 480 | 1692 ± 29.7 | 105 |
|  |  |  |  | 1200 | 2511 ± 16.3 | 110 |
| **Isovaleric acid** | 3 | 15 | 3851 ± 335 | 462 | 4332 ± 23.3 | 104 |
|  |  |  |  | 2308 | 6222 ± 250 | 103 |
|  |  |  |  | 4615 | 8465 ±549 | 99.9 |
| **Valeric acid** | 2 | 7 | 23 ± 0.0 | 58 | 81.5 ± 2.2 | 101 |
|  |  |  |  | 233 | 255 ± 21.2 | 99.8 |
|  |  |  |  | 465 | 537 ± 55.2 | 110 |
| **4-Methylvaleric acid** | 3 | 7 | 13.5 ± 2.1 | 58 | 78 ± 2.8 | 112 |
|  |  |  |  | 231 | 260 ± 9.9 | 107 |
|  |  |  |  | 462 | 514 ± 12.7 | 108 |
| **Hexanoic acid** | 2 | 8 | 30 ± 5.7 | 58 | 89.5 ± 2.1 | 102 |
|  |  |  |  | 232 | 274 ± 6.4 | 105 |
|  |  |  |  | 465 | 533 ± 6.4 | 108 |

**Table S4** Intra-day and inter-day precision and trueness

| **Target Compound** | **Concentration (µg/mL)** | **Intra-day precision (%CV, n=6)** | **Intra-day trueness (% bias, n=6)** | **Inter-day precision (%CV, n=6)** | **Inter-day trueness (% bias, n=6)** |
| --- | --- | --- | --- | --- | --- |
| **Acetic acid** | 0.75 | 6.37 | -4.19 | 6.01 | -1.01 |
|  | 1.5 | 7.03 | -2.80 | 4.59 | -13.31 |
|  | 2.5 | 5.99 | 11.27 | 11.44 | 9.99 |
|  | 5 | 6.99 | 18.75 | 5.37 | 13.92 |
|  | 10 | 12.58 | 9.19 | 11.75 | 7.79 |
|  | 20 | 5.96 | 0.16 | 1.21 | -1.43 |
|  | 25 | 4.25 | 0.78 | 5.39 | -3.69 |
| **Propionic acid** | 0.075 | 6.40 | 10.29 | 3.77 | 10.77 |
|  | 0.15 | 2.81 | 8.41 | 2.57 | 12.14 |
|  | 0.25 | 5.72 | 23.29 | 7.72 | 10.48 |
|  | 0.5 | 3.43 | -17.01 | 4.96 | -10.17 |
|  | 1 | 2.36 | 1.55 | 2.76 | 11.57 |
|  | 2 | 2.57 | 2.23 | 2.60 | 4.50 |
|  | 2.5 | 0.82 | 1.65 | 1.90 | 2.38 |
| **Isobutyric acid** | 0.075 | 0.48 | 7.75 | 1.31 | 8.43 |
|  | 0.15 | 0.77 | -0.78 | 0.34 | -0.82 |
|  | 0.25 | 0.57 | -7.43 | 0.67 | -7.72 |
|  | 0.5 | 3.58 | 1.39 | 3.19 | -0.29 |
|  | 1 | 1.62 | 1.26 | 0.92 | 1.14 |
|  | 2 | 4.78 | -2.29 | 4.19 | -3.53 |
|  | 2.5 | 0.74 | 0.46 | 0.88 | 0.74 |
| **Butyric acid** | 0.075 | 4.86 | -6.17 | 5.75 | -3.57 |
|  | 0.15 | 1.91 | -6.13 | 1.71 | -6.32 |
|  | 0.25 | 5.77 | 1.91 | 4.97 | 12.70 |
|  | 0.5 | 6.98 | -0.34 | 3.64 | 1.68 |
|  | 1 | 4.74 | 6.81 | 2.25 | 1.91 |
|  | 2 | 7.65 | -6.24 | 7.97 | -3.19 |
|  | 2.5 | 6.67 | 5.34 | 4.76 | -3.36 |
| **Isovaleric acid** | 0.3 | 2.92 | 8.14 | 5.95 | 11.68 |
|  | 0.6 | 5.48 | 19.81 | 8.49 | 14.18 |
|  | 1 | 3.06 | -1.36 | 3.71 | 2.75 |
|  | 2 | 2.73 | 2.11 | 3.87 | 6.43 |
|  | 4 | 3.97 | -1.79 | 4.29 | 4.47 |
|  | 8 | 5.22 | 1.89 | 5.93 | 6.35 |
|  | 10 | 4.27 | 2.27 | 5.11 | 4.48 |
| **Valeric acid** | 0.03 | 3.66 | 8.80 | 4.20 | 14.07 |
|  | 0.06 | 5.67 | 14.23 | 3.03 | 10.37 |
|  | 0.1 | 2.26 | -13.48 | 1.62 | -8.85 |
|  | 0.2 | 3.49 | 0.93 | 1.60 | 1.23 |
|  | 0.4 | 7.20 | -4.75 | 5.08 | 8.54 |
|  | 0.8 | 4.20 | -0.94 | 5.80 | 0.21 |
|  | 1 | 2.97 | -1.37 | 3.39 | 2.56 |
| **4-Methylvaleric acid** | 0.03 | 1.89 | -19.49 | 2.87 | -18.63 |
|  | 0.06 | 1.96 | -10.92 | 1.89 | -12.50 |
|  | 0.1 | 10.63 | -19.76 | 5.95 | -7.00 |
|  | 0.2 | 3.42 | 15.48 | 3.84 | 11.64 |
|  | 0.4 | 1.89 | 2.17 | 3.14 | -0.22 |
|  | 0.8 | 3.87 | 2.27 | 5.99 | 1.85 |
|  | 1 | 3.59 | -3.61 | 3.61 | -4.82 |
| **Hexanoic acid** | 0.03 | 3.07 | 2.07 | 2.29 | -6.53 |
|  | 0.06 | 2.30 | 7.58 | 2.22 | 7.92 |
|  | 0.1 | 2.43 | 8.07 | 2.58 | 14.88 |
|  | 0.2 | 4.54 | 9.30 | 3.34 | -10.94 |
|  | 0.4 | 2.12 | 3.51 | 5.18 | 11.97 |
|  | 0.8 | 2.17 | -1.79 | 2.56 | 2.52 |
|  | 1 | 8.21 | 12.43 | 5.39 | 1.12 |

**Table S5** Comparison of PRM mode in LC-HRMS and MRM mode in LC-QQQ for analysis of SCFAs in five pooled serum samples. SCFAs were separated on an ACE C18_AR column on both instruments.

| **Analyte** | **Pool serum 1 (ng mL^-1^)** | | |  | **Pool serum 2 (ng mL^-1^)** | | |  | **Pool serum 3 (ng mL^-1^)** | | |  | **Pool serum 4 (ng mL^-1^)** | | |  | **Pool serum 5 (ng mL^-1^)** | |  |
| --- | --- | --- | --- | --- | --- | --- | --- | --- | --- | --- | --- | --- | --- | --- | --- | --- | --- | --- | --- |
|  | **PRM** | **MRM** | **Agreement**  **(%)** |  | **PRM** | **MRM** | **Agreement**  **(%)** |  | **PRM** | **MRM** | **Agreement**  **(%)** |  | **PRM** | **MRM** | **Agreement**  **(%)** |  | **PRM** | **MRM** | **Agreement**  **(%)** |
| Acetic acid | 14156 | 14957 | 94.6 |  | 14000 | 14198 | 98.6 |  | 12598 | 12477 | 101 |  | 11781 | 12053 | 97.7 |  | 11290 | 11297 | 99.9 |
| Propionic acid | 1283 | 1311 | 97.9 |  | 1223 | 1219 | 100 |  | 1080 | 1026 | 105 |  | 1110 | 1093 | 102 |  | 786 | 756 | 104 |
| Isobutyric acid | 1749 | 1699 | 103 |  | 1747 | 1652 | 106 |  | 1471 | 1463 | 100 |  | 1291 | 1289 | 100 |  | 917 | 905 | 101 |
| Butyric acid | 1383 | 1356 | 102 |  | 1504 | 1312 | 115 |  | 1219 | 1220 | 100 |  | 1285 | 1275 | 101 |  | 247 | 211 | 118 |
| Isovaleric acid | 2801 | 2750 | 102 |  | 3333 | 3307 | 101 |  | 2987 | 3152 | 94.8 |  | 3221 | 3322 | 97 |  | 59 | 51 | 116 |
| Valeric acid | 108 | 117 | 92.3 |  | 104 | 117 | 88.9 |  | 80.0 | 102 | 78.4 |  | 82.0 | 105 | 78.1 |  | 68 | 90 | 75.6 |
| 4-Methylvaleric acid | 15 | 13 | 115 |  | 17 | 13 | 131 |  | 13 | 16 | 81.2 |  | 17 | 13 | 131 |  | 16 | 14 | 114 |
| Hexanoic acid | 821 | 822 | 100 |  | 725 | 646 | 112 |  | 631 | 798 | 79.1 |  | 703 | 733 | 95.9 |  | 344 | 360 | 95.6 |

**Proposed elemental composition and MS/MS pattern for SCFA interferences**

**Acetic acid interference**

proposed elemental composition: C_8_H_8_O_3_N_3_

MS/MS pattern


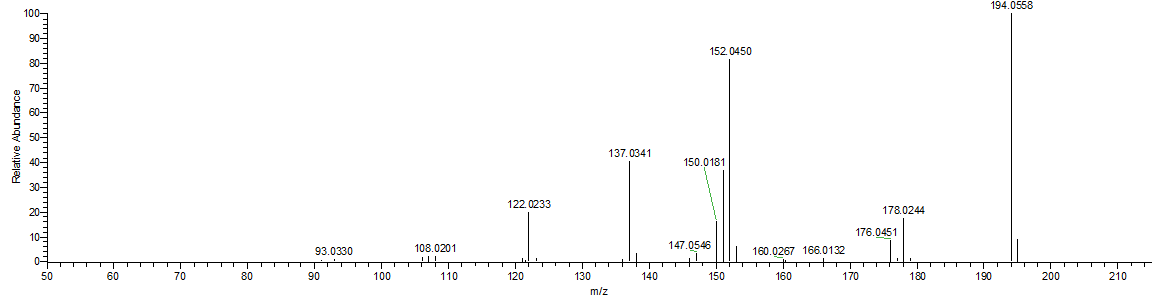


**Isobutyric/butyric acid interferences**

proposed elemental composition: C_5_H_11_O_5_N_5_; C_6_H_7_ON_9_; C_7_H_13_O_6_N_2_; C_3_H_9_O_4_N_8_; C_8_H_9_O_2_N_6_; C_10_H_11_O_3_N_3_

MS/MS pattern

**
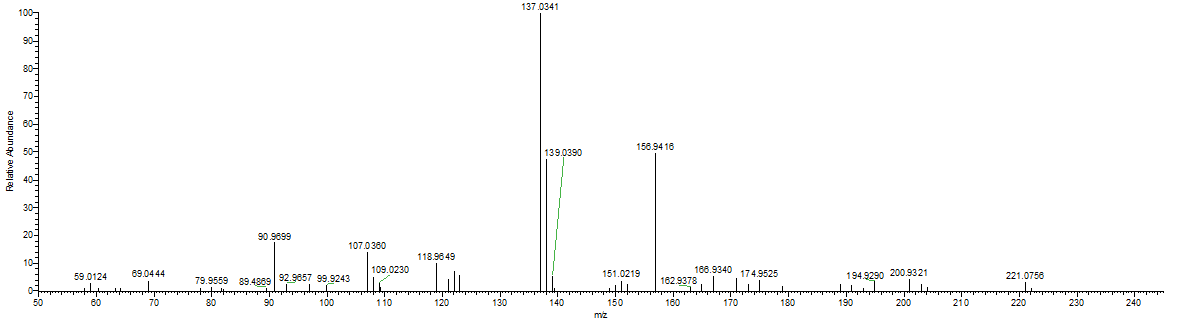
**

**Isovaleric/valeric acid interference**

Proposed elemental composition: C_11_H_14_O_3_N_3_

MS/MS


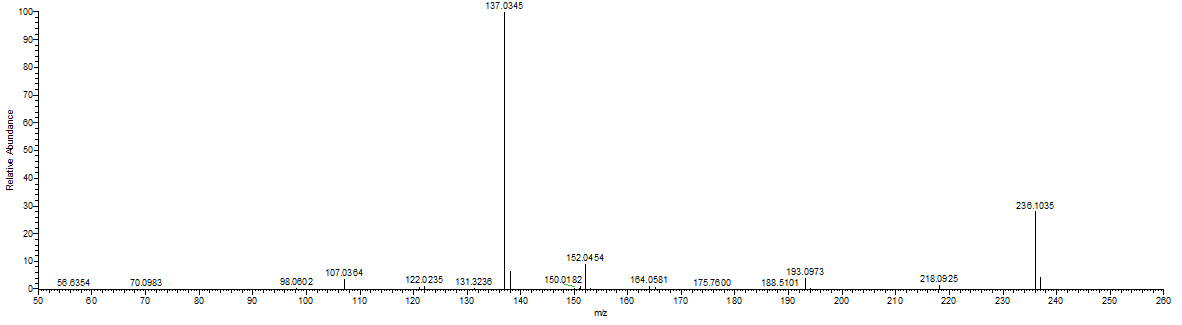


**4-Methyl valeric acid interferences**

Proposed elemental composition: C_12_H_16_O_3_N_3_

MS/MS pattern


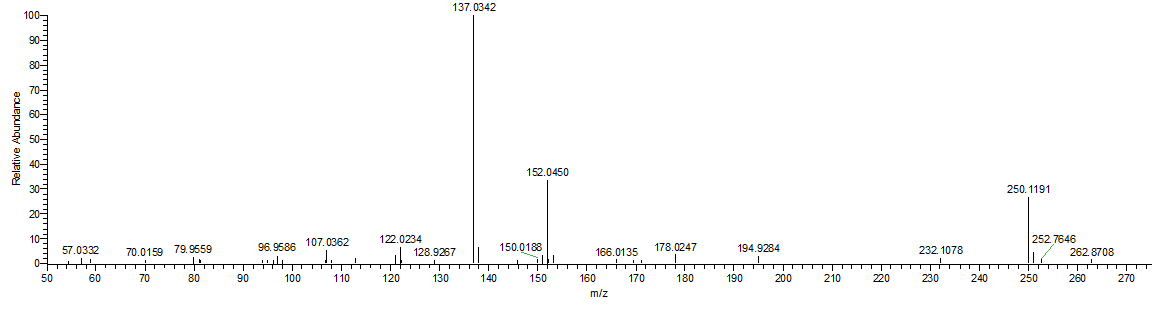


**4-Methyl valeric acid interferences**

Proposed elemental composition: C_17_H_18_N_2_; C_6_H_18_O_3_N_8_; C_14_H_20_O_3_N

MS/MS pattern


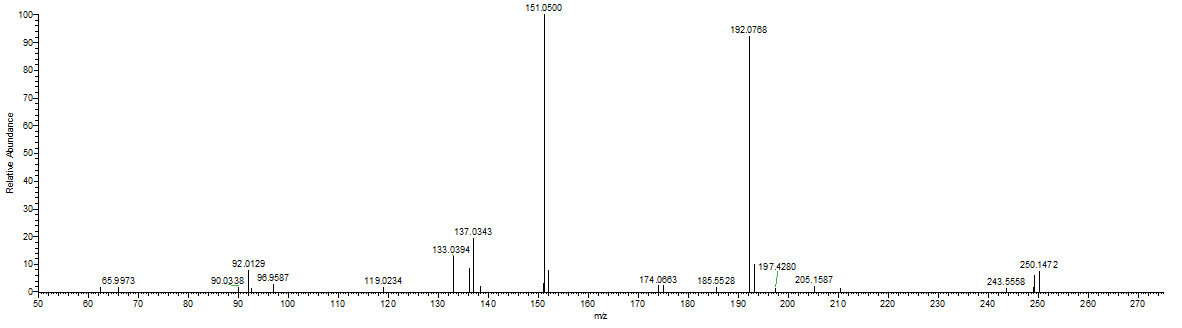

Supplement: Supplementary file 1 — (DOCX 165 kb) [file 216_2021_3589_MOESM1_ESM.docx]
